# HEART-FAILURE TRAINING FOR HOME CARE WORKERS IN NEW YORK CITY

**DOI:** 10.1093/geroni/igz038.792

**Published:** 2019-11-08

**Authors:** Madeline Sterling, Peggy Leung

**Affiliations:** Weill Cornell Medical College, New York, New York, United States

## Abstract

Home care workers (HCWs), which include home health aides and personal care aides, are increasingly being used by community dwelling adults with heart failure (HF) for long-term assistance and post-acute care. Findings from our prior research suggest that HCWs are deeply involved in many aspects of HF patients’ self-care, including HF maintenance and management, but the majority have not received any HF training or HF-specific resources. Due to this, many HCWs do not feel confident caring for their clients with HF. In this symposium, we will present the findings of a qualitative study that used a nominal group technique to elicit the educational needs of 40 English and Spanish speaking agency-employed HCWs caring for HF patients in New York City. We will also present an overview of the HF training course that was developed from this data and its effect on HCWs’ HF knowledge and caregiving self-efficacy.

